# 42. Outbreak of SARS-CoV-2 in Hospitalized Hemodialysis Patients: an Epidemiologic and Genomic Investigation

**DOI:** 10.1093/ofid/ofab466.042

**Published:** 2021-12-04

**Authors:** Charles E Marvil, Anne Piantadosi, Aaron Preston, Andrew Webster, Jeannette Guarner, Kari L Love, Susan Ray, Paulina Rebolledo, Yun Wang, Jesse Waggoner, Jesse T Jacob, Ahmed Babiker

**Affiliations:** 1 Emory University School of Medicine, Decatur, GA; 2 Emory University, Atlanta, Georgia; 3 Emory University Midtown Hospital, Decatur, Georgia; 4 Emory University School of Medicine and Georgia Emerging Infections Program, Atlanta, Georgia; 5 Emory Healthcare, Atlanta, Georgia; 6 Emory University School of Medicine, Emory University Rollins School of Public Health, Atlanta, GA; 7 Emory University School of Medicine, Grady Memorial Hospital, Atlanta, GA

## Abstract

**Background:**

Healthcare-associated transmission of SARS-CoV-2 is relatively rare and may be difficult to quantify. We performed an epidemiological investigation and SARS-CoV-2 genome sequencing to define the source and scope of a SARS-CoV-2 outbreak in a cluster of hospitalized patients

**Methods:**

We conducted an outbreak investigation after identifying hospital-onset COVID-19 in patients receiving hemodialysis in January 2021. Electronic medical record review, staff interviews, review of employee schedule logs, and contact tracing were used to determine the outbreak timeline and identify exposed healthcare workers (HCW). SARS-CoV-2 genomes were sequenced from residual nasopharyngeal swab samples from 6 individuals in the outbreak investigation and compared to sequences from 14 patients in the same facility, 54 patients in nearby facilities, and 375 publicly available sequences from individuals in the state of Georgia.

**Results:**

Eight patients with hospital-onset COVID-19 were identified (Cases 1-8); all were receiving hemodialysis and 5 were bedded in a single inpatient nursing unit. Among 53 potentially exposed HCW, 29 underwent testing and 5 were positive (Cases 9-13). The suspected index patient (Case 1) was found to have been coughing and inconsistently wearing a mask during a hemodialysis session on the same day that 6 of the 7 other patients and one HCW (Case 10) were in close proximity in the hemodialysis unit (**Figure 1A**). Further investigation revealed lack of use of curtain barriers in the hemodialysis bays, inconsistent use of personal protective equipment by HCW, and overcrowding of staff breakrooms. Among the 6 samples available for phylogenetic analysis, SARS-CoV-2 sequences from 5 (4 patients and 1 HCW, Case 9) were identical and at least 4 SNPs removed from the next closest sequence in this study, supporting a transmission cluster (**Figure 1B**). The sequence from the sixth sample (HCW Case 10) was phylogenetically distinct, indicating an independent source of infection.

Figure 1

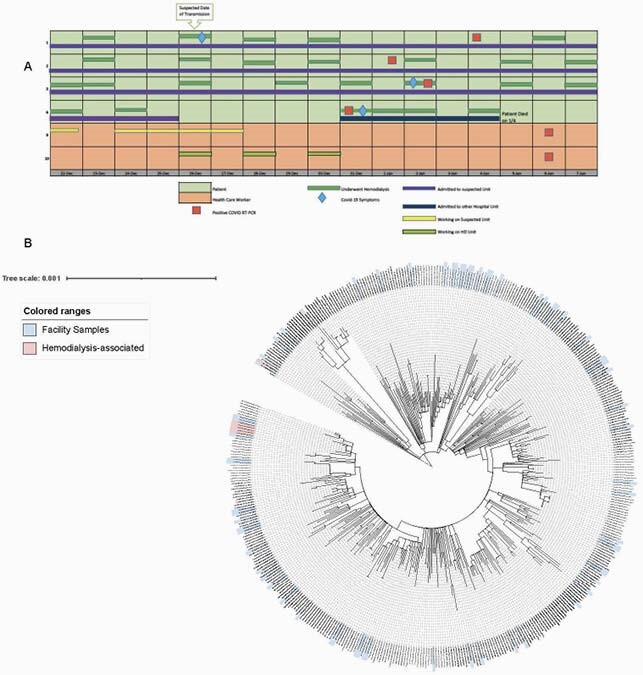

Exposure and onset of symptoms for the 6 cases in the outbreak with samples available for SARS-CoV-2 sequencing. Four patients with hospital-onset COVID-19 (Cases 1-4) were receiving hemodialysis and bedded in a single inpatient nursing unit, with two exposed healthcare workers (Cases 9-10). (A). Phylogenetic tree of SARS-CoV-2 genomes from individuals in this outbreak investigation (red), as well as 14 patients in the same facility and 54 patients in nearby facilities between 12/12/2020 and 1/13/2021 (blue). These were aligned with 375 publicly available sequences from individuals in the state of Georgia from the same time period using MAFFT. A maximum-likelihood phylogenetic tree was generated under a generalized time-reversible model with 1,000 bootstrap replicates using IQtree v2.0.3 and visualized and annotated using Interactive Tree of Life (iTOL) v4 (B).

**Conclusion:**

Lack of appropriate respiratory hygiene led to SARS-CoV-2 transmission during a single hemodialysis session, based on clinical and genomic epidemiology. Use of appropriate PPE for both patients and HCW and other infection prevention measures are critical to prevent SARS-CoV-2 transmission.

**Disclosures:**

**All Authors**: No reported disclosures

